# Could low-acuity emergency medical services patients be redirected to primary care? Findings from a multi-center survey in Berlin, Germany

**DOI:** 10.1186/s12873-025-01295-9

**Published:** 2025-07-30

**Authors:** Felix Holzinger, Lisa Kümpel, Rebecca Resendiz Cantu, Anja Alberter, Martin Möckel, Christoph Heintze

**Affiliations:** 1 Charité – Universitätsmedizin Berlin, corporate member of Freie Universität Berlin and Humboldt-Universität zu Berlin, Institute of General Practice, Charitéplatz 1, 10117 Berlin, Germany; 2 Charité – Universitätsmedizin Berlin, corporate member of Freie Universität Berlin and Humboldt-Universität zu Berlin, Division of Emergency Medicine, Campus Mitte and Virchow, Charitéplatz 1, 10117 Berlin, Germany

**Keywords:** Emergency department, General practitioner, Emergency medical services, Preclinical redirection, Health services research

## Abstract

**Background:**

Emergency medical services (EMS) are frequently used by low-acuity patients, which contributes to emergency department (ED) crowding. The feasibility of EMS transporting low-acuity patients directly to general practitioner (GP) practices remains a matter of debate. We therefore investigated the circumstances of EMS utilization in patients who subsequently receive ambulatory treatment in the ED. We wanted to find out how often a primary care (PC) consultation could have been a suitable alternative in such cases.

**Methods:**

Low-acuity ED utilizers transported by EMS were surveyed on demographics and medical characteristics and asked about the appropriateness and acceptability of a potential PC redirection, supplemented with case assessments by EMS personnel. Additionally, treatment documentation from both the ED and EMS was analyzed. Descriptive statistics were conducted. Associations between categorical variables were examined by Chi^2^ tests.

**Results:**

A total of *n* = 358 low-acuity EMS participants were recruited. Participants had a mean age of 47.6 years; gender f/m: 58.1%/41.9%. In the hospital, 71.8% were assigned to the Manchester triage system (MTS) category 3 and 28.0% to category 4. A third of the patients had decided to alert EMS at their discretion, while other people (e.g., relatives, colleagues) had been involved twice as often. Patients most commonly cited severe symptoms and related fears as reasons for engaging EMS services. EMS personnel categorized the complaints as treatable by a GP in 37.0%, while 44.5% of patients would have been open to PC management. However, these assessments exhibited substantial discrepancies, as evidenced by a Cohen’s Kappa coefficient of approximately 0.1. From a preclinical perspective, only 24.4% of cases met the criteria for potentially realistic diversion. These encompassed both patient openness to alternative care pathways and EMS discernment of cases as potentially appropriate.

**Conclusions:**

PC diversion is estimated to be feasible for a maximum of a quarter of ED outpatients. Markers for potential management in PC show highly discrepant results, and there is no validated system or score for preclinical identification of patients eligible for safe redirection. As EMS is intended for high-acuity emergencies, such patients could potentially also benefit from options like telemedicine care at home or alternative transportation.

**Trial registration:**

German Clinical Trials Register (DRKS00023480); date: 27/11/2020.

## Background

Emergency medical services (EMS) are frequently utilized by patients with low-acuity conditions, increasing EMS caseload [1] and potentially aggravating emergency department (ED) crowding [2, 3]. The strain on EMS and EDs has prompted discussions about the potential for redirecting low-acuity patients to more adequate primary care (PC) settings, alleviating pressure on EDs and EMS and optimizing patient care [4–6]. A respective debate was initiated in German national healthcare policy in 2018, when the Advisory Council on the Assessment of Developments in the Health Care System – the expert panel tasked with providing the government with recommendations for health care reform – suggested optional direct redirection of low-acuity EMS cases to routine ambulatory care services, e.g., to general practitioners (GP), to avoid unnecessary ED visits [7].

However, the feasibility and acceptance of such redirections remain a subject of controversial debate [6]. Previous studies have shown that while GP-level care may be adequate and beneficial for certain patients, their subjective perception of what constitutes an emergency varies widely, frequently leading to ED utilization [8–10]. The EMAPREPARE study thus aimed to investigate the potential for redirection of low-acuity EMS patients in a mixed methods design.

In a previously published investigation, we had presented findings from the study’s qualitative module, which consisted of interviews with EMS personnel about their views and perceptions regarding feasibility and acceptance of redirection [11]. Complementary, this paper reports on the quantitative part of the study, which aimed to investigate the specific circumstances under which EMS are utilized by low-acuity patients who ultimately receive ambulatory treatment at the ED. Additionally, we wanted to assess how often a GP consultation could realistically be an alternative to an ED visit for these patients by collating data from patient and EMS personnel surveys and hospital documentation.

## Methods

### Research network and study setting

This observational study was conducted in the Berlin research network EMANet (Emergency and Acute Medicine Network for Health Care Research Berlin). Findings of the qualitative interviews with EMS personnel accompanying the quantitative study results presented in this paper have already been published [11].

### Recruitment and data collection

Patients were recruited from three EDs in central Berlin between June 2021 and January 2024. Low-acuity ED consulters who were transported to the ED by EMS were eligible to take part in the study. Low-acuity was defined as triaged in categories three to five of the five-stepped Manchester Triage System (MTS) [12] and outpatient treatment at the ED. Additional inclusion criteria were a minimum age of 18 years and the ability to give formal informed consent to participate. Recruitment was conducted during standard practice hours on weekdays (Monday through Friday, 8:00–18:00) to reflect conditions under which PC diversion could be theoretically implemented.

After consent, patients were surveyed by study personnel while waiting in the ED. Patients admitted to inpatient care were excluded post hoc from the study, as the final disposition was not decided at the time of the survey. The quantitative questionnaire consisted of 40 items on demographics, medical characteristics (e.g., general health, mental health, chronic conditions), health care utilization (HCU), subjective urgency, as well as motives for ED consultation. The questionnaire included both validated instruments and custom-designed items that had been successfully used in preceding EMANet surveys [13, 14].

Migration history was defined as not being born in Germany and not being a tourist. Education status was scaled according to the CASMIN (Comparative Analysis of Social Mobility in Industrial Nations) classification [15]. Chronic conditions were inquired about by a comprehensive multiple-choice checklist. For general health, a five-point Likert scale was used (scale: ‘very good’ to ‘very bad’) [16], and general life satisfaction was measured by the short scale L-1 [17]. For mental health, the PHQ-4 instrument was used (possible values of 0–12 and sub-scores of 0–6 for anxiety and depression) [18], and data on HCU were collected through questions based on the German Health Interview and Examination Survey for Adults [19].

Concurrently, EMS personnel having conveyed the respective patients to the ED were surveyed about the cases and asked about their opinion on a potential alternative treatment in PC. The short questionnaire consisted of only four questions in order not to unnecessarily disrupt EMS turnaround times and maximize participation.

Diagnoses were derived from ED documentation, and NACA-S (National Advisory Committee for Aeronautics) scores of EMS severity judgment [20] were extracted from preclinical EMS documentation. NACA-S categories I and II represent light to moderate conditions potentially not needing inpatient care, while III and IV are assigned to more severe conditions that may likely require on-site emergency physician treatment and inpatient management.

For post hoc determination of cases that could potentially have been managed in PC, ED diagnoses and procedures were evaluated, and cases filtered accordingly.

### Data preparation and analysis

Most data were used for analysis as collected. The CASMIN education scale was trichotomized into low, intermediate, and high educational attainment for reporting. For Likert scale data on self-reported general health, data of the upper two categories (‘very good’ / ‘good’) and lower two categories (‘bad’ / ‘very bad’) were combined. Motives for EMS utilization were thematically grouped into summary categories as done in previous reports [13], based on the Coster et al. framework [21]. For some questions in the survey, multiple answers were possible; thus, there is an overlap between categories as implicit in multi-response data.

Descriptive statistics were performed. Furthermore, associations between categorical variables were investigated statistically by Chi^2^ tests and calculation of Phi coefficients.

To estimate realistic diversion potential from a patient-centered viewpoint, we combined data from the patient survey and the EMS survey. As prerequisites to achieving a successful PC redirection, we considered both acceptance by the patient (openness to be diverted) and EMS judgment as potentially manageable by a GP. For assessment of inter-rater agreement of patient and EMS personnel views, Cohen’s Kappa coefficient was used.

As to post hoc categorization of patient eligibility for alternative GP care based on ED documentation, cases were considered non-eligible if their diagnoses, procedures performed, or monitoring requirements suggested the necessity of a hospital setting for adequate assessment and treatment. This applied to the following cases:

- Investigation for possible fractures, joint dislocations, head trauma, spinal trauma, or stroke with imaging performed.
- Injury requiring more extensive surgical care, e.g., partial amputations.
- Victims of violent crime, intoxication, electrical accidents.
- Any patient investigated with computed tomography or magnetic resonance imaging.

All data were analyzed in SPSS Version 29 (IBM Corp., Armonk, NY, USA) and R 4.1.2 (R Foundation for Statistical Computing, Vienna, Austria). For all analyses, a significance level of 0.05 was set.

## Results

### Population characteristics

Between June 2021 and January 2024, a total of *n* = 358 patients were included. Demographic, health-related, and consultation-specific data of the study cohort is summarized in Table 1.

**Table 1:** Characteristics of study participants

Variable	Measure	Total cohort	
**Participants**	n	358	
***Demographics***			
**Age**	n	358	
	Mean (SD) Median (Range)	47.6 (20.1) 46.0 (18–96)	
**Sex**	n	358	
Male	n, %	150	41.9
Female	n, %	208	58.1
**Migration and travel**	n	351	
Migrant (first generation)	n, %	60	17.1
Tourist	n, %	16	4.6
**Education (CASMIN)**	n	347	
Low	n, %	62	17.9
Intermediate	n, %	153	44.1
High	n, %	132	38.0
**Living situation**	n	353	
Single household	n, %	135	38.2
**Social situation**	n	351	
Steady partnership	n, %	196	55.8
***ED symptoms***			
**Triage category**	n	354	
3	n, %	254	71.8
4	n, %	99	28.0
5	n, %	1	0.3
**Symptom duration**	n	350	
Started today	n, %	262	74.9
Started yesterday	n, %	20	5.7
Several days, but < 1 week	n, %	32	9.1
> 1 week	n, %	36	10.3
**Symptom-associated distress**	n	357	
	Mean (SD) Median (Range)	6.3 (2.4) 7.0 (0–10)	
**Subjective urgency: I must be seen…**	n	343	
immediately	n, %	57	16.6
as soon as possible	n, %	121	35.3
today	n, %	105	30.6
not as urgently	n, %	60	17.5
***Health***			
**Morbidity**	n	349	
Any chronic condition	n, %	216	61.9
≥ 2 chronic conditions	n, %	159	45.6
≥ 3 chronic conditions	n, %	117	33.5
**PHQ-4 anxiety subscale**	n	351	
	Mean (SD) Median (Range)	1.3 (1.5) 1.0 (0–6)	
**PHQ-4 depression subscale**	n	352	
	Mean (SD) Median (Range)	1.3 (1.6) 1.0 (0–6)	
**General health**	n	358	
Very good / good	n, %	225	62.8
Average	n, %	102	28.5
Bad / very bad	n, %	31	8.6
**General life satisfaction**	n	357	
	Mean (SD) Median (Range)	7.3 (2.0) 8.0 (0–10)	

Note. n = cases with available data for respective characteristic; % = percentage of cases with available data; Ranges reported with median values refer to minimum and maximum; Sex: self-reported; Migration and travel: Migrant = not born in Germany, not a tourist; Education: CASMIN (Comparative Analysis of Social Mobility in Industrial Nations) scale, trichotomized; Triage category: Manchester Triage system (MTS); Subjective symptom-associated distress, general life satisfaction: 0–10 scales; PHQ-4 anxiety and depression: 0–6 subscales; General health: 5-point Likert scale

The average age of the study population was 47.6 years. More than 60% of the patients had at least one chronic condition, but just as many stated that they considered their general state of health to be ‘good’ or ‘very good’. Correspondingly, general life satisfaction had a mean score of 7.3, with a median of 8, suggesting a relatively high level of life satisfaction.

As shown in Table 1, 71.8% were categorized as triage level 3, indicating a higher priority, while 28% fell into category 4, and a minimal 0.3% were in category 5, representing the lowest urgency. Symptom onset was on the same day in most cases. Subjective symptom-associated distress level was moderate to high across the cohort. 51.9% of patients stated that they need to be seen immediately or as soon as possible and only 17.5% of the patients believed that their condition was ‘not as urgent’, equivalent to not needing a same-day evaluation.

Preclinically, NACA-S categories suggesting potential ambulatory management (I or II), were assigned to 73.5% of cases (data for *n* = 339). Nevertheless, as per our inclusion criteria, all patients in the cohort had been managed as outpatients in the ED, including the cases initially assigned a NACA-S score of III or IV.

ED diagnoses extracted from hospital records indicated that consultations were most frequently caused by musculoskeletal complaints or minor trauma (44.4%, *n* = 354 cases with data), cardiovascular symptoms (16.4%) and neurological symptoms (14.7%).

### Health care utilization

89.7% of all patients reported having a GP, and 72.3% reported at least one GP consultation in the past six months, with an average of 2.9 visits. Additionally, 67.3% had consulted a specialist during the same period, with an average of 4.1 visits. Only 5.3% of patients contacted their GP before calling EMS. Attempts to contact the Statutory Health Insurance Physicians’ ambulatory emergency service prior to alerting EMS were reported by 4.5%. However, the outcomes of these consultations were not explored in the survey. Only a minority reported repeat EMS use (15.1% in the past six months). Lastly, 22.3% had visited an ED in the past six months, averaging 1.7 visits (Table 2).

**Table 2:** Patients’ health care utilization

Variable	Measure	Total cohort	
**Participants**	n	358	
**Utilization of GP care**			
Has a GP	n, %	321	89.7
Visited GP in the past 6 months	n, %	259	72.3
Number of GP visits in the past 6 months (data for *n* = 242)	Mean (SD) Median (Range)	2.9 (4.2) 2.0 (1–50)	
**Utilization of specialist care (apart from GP)**			
Visited specialist in the past 6 months	n, %	241	67.3
Number of specialists visits in the past 6 months (data for *n* = 230)	Mean (SD) Median (Range)	4.1 (4.8) 3.0 (1–43)	
**Utilization of EMS**			
Used EMS in the past 6 months	n, %	54	15.1
Number of EMS uses in the past 6 months (data for *n* = 53)	Mean (SD) Median (Range)	1.9 (1.7) 1 (1–11)	
**Utilization of ED**			
Visited ED in the past 6 months	n, %	80	22.3
Number of ED uses in the past 6 months (data for *n* = 77)	Mean (SD) Median	1.7 (1.3) 1 (1–6)	

Note. Not counting today’s utilization/contact; utilization means/medians for utilizers of respective provider category only

### Motives for using EMS

Participants were asked about their motives and decision-making regarding their alerting EMS. The most frequent reasons concerned symptom-associated anxiety and the perceived severity of the acute health condition, reported by 67.6% of participants. Other important aspects voiced were the subjective need for rapid help (31.0%), EMS being alerted by people other than the patient (28.2%), and personally seeing or knowing no adequate alternative (7.3%). The decision to contact EMS was most commonly related as the patients’ own (34.1%), followed closely by relatives or friends (33.8%), and by passersby or strangers (22.1%) (Table 3).

**Table 3:** Patients’ motives and context of EMS utilization

Variable	Measure	Total cohort	
**Participants**	n	358	
**Grouped Motives for contacting EMS**			
Fear / severity	n, %	242	67.6
Access	n, %	18	5.0
Speed	n, %	111	31.0
Convenience	n, %	7	2.0
Prior good experiences with EMS/ED	n, %	8	2.2
Know no alternative	n, %	26	7.3
EMS was alerted by another person	n, %	101	28.2
**Who took the decision to contact EMS?**			
Patient	n, %	122	34.1
Passer-by / stranger	n, %	79	22.1
Relatives, friends, acquaintances, work colleagues	n, %	121	33.8
Health care professional or nursing home	n, %	8	2.2
Somebody else	n, %	25	7
No information	n, %	2	0.6

Note. Motives for using ED: Multi-response data with category overlap; thus, percentages cannot be added up. Category ‘fear / severity’ encompasses reasons related to the severity of symptoms and to health-related anxiety, whereas ‘speed’ summarized reasons related to the need for timely help. ‘Convenience’ reasons concern e.g., ambulance transport being cheaper than a taxi or waiting times presumedly shorter. Other summary categories are self-explanatory

### Case assessment: redirection and GP treatment

Both patients and the respective transporting EMS personnel were asked to assess the potential for PC management. When presented with the concept, about 56.2% of the surveyed patients (data for n = 322) endorsed the idea of diverting appropriate cases with acute, low-acuity health conditions to GP practices. This was indicated by responses in the highest two categories of a Likert scale (‘applies’, or ‘applies fully’).

When inquired on a personal level, 44.5% of patients would have been open to GP treatment instead of the ED in their specific situation, but only 15.1% believed that their specific problem could have been adequately managed in a PC setting. The most frequently stated reason for this assessment concerned the presumedly limited resources of GP practices regarding diagnostics and treatment (64.1% of *n* = 231). Patients’ openness to diversion and assessment of the appropriateness of the setting were moderately correlated (Phi 0.390, *p* < 0.001, Chi^2^ test, *n* = 226 cases with an answer of either ‘yes’ or ‘no’ for both items).

EMS personnel classified the patients’ complaints as manageable by a GP in 36.9% of cases. However, when comparing the ratings at the case level, assessments differed considerably with a Cohen’s Kappa coefficient for inter-rater reliability of only 0.102 (*n* = 275), indicating low agreement. The most important reason for patients not judging GP management as adequate was a presumed lack of diagnostic and therapeutic capacities in the PC setting (Table 4).

When asked why GP treatment would not have been suitable for the patient in question, EMS personnel most frequently judged the patient’s symptoms as too complex to investigate adequately in a GP practice (e.g., as to lack of diagnostic capabilities like X-ray), reflecting the concerns voiced in the patient questionnaire. Further important reasons related to the patient’s immobility or a severe health condition, presumably requiring a hospital setting (Table 4).

Considering ED diagnoses, data showed that EMS personnels’ judgment as potentially GP treatable was significantly less likely if the complaints were musculoskeletal, e.g., minor trauma (*p* = 0.011, Chi^2^ test, *n* = 353 cases). There were no significant associations with other complaint classifications. Considering demographic markers, EMS judgment as a potential GP case was significantly more frequent in first generation migrants (*p* = 0.007, Chi^2^ test, *n* = 357 cases), while there was no statistical association with age or sex.

**Table 4:** Case assessment by patients and EMS personnel: Redirection and potential GP treatment

Variable		Measure	Total cohort	
**Case assessment by patient: appropriateness of PC setting and openness to diversion**				
**Cases with data**		n	357	
Could a GP have managed your problem?	Yes	n, %	54	15.1
	No	n, %	231	64.7
	Undecided	n, %	72	20.2
Would you have been prepared to be treated at a GP practice as an alternative to EMS?	Yes	n, %	159	44.5
	No	n, %	117	32.8
	Undecided	n, %	81	22.7
**Patient-reported reasons why a GP could presumably not have managed the problem**				
**Medical reasons**	Symptoms are too severe	n, %	47	20.3
	GP is not competent enough	n, %	51	22.1
	Not able to reach GP on time	n, %	40	17.3
**Structural reasons**	GP does not offer needed diagnostics or treatment	n, %	148	64.1
	Time resources at GP are not sufficient	n, %	23	10.0
	Immobile, unable to reach GP	n, %	53	22.9
	Out of practice opening hours	n, %	9	3.9
**Case assessment by EMS personnel: appropriateness of PC setting**				
Cases with data		n	358	
Could the patient have been managed in a GP practice?	Yes	n, %	132	36.9
	No	n, %	225	62.8
	Undecided	n, %	1	0.3
**EMS-reported reasons why a GP could presumably not have managed the problem**				
**Medical reasons**	Symptoms are too severe	n, %	50	22.2
**Structural reasons**	Patient immobile	n, %	72	32.0
	GP does not offer needed diagnostics or treatment / too complex for investigation by GP	n, %	167	64.2

Note. n = number of participants with data for item; multi-response data for patient- and EMS-reported reasons why GP could have not managed the problem, with inherent category overlap. Percentages reported for reasons refer to the total of participants answering ‘no’ when asked whether a GP could have managed the problem

Concerning realistic diversion potential, only 24.4% of cases (*n* = 67 of *n* = 275 cases with available data) met the criteria of both patient openness to being diverted and EMS judgment as potentially appropriately manageable in PC.

In the post hoc analysis of the hospital records (ED documentation) of these patients, *n* = 272 (76.0%) did not show a criterion suggesting ineligibility for PC management, while *n* = 86 (24.0%) did so (see methods section for criteria list). Being classified as a potential PC case based on diagnoses and procedures performed was neither statistically associated with the triage category (*p* = 0.610, Chi^2^ test, *n* = 353 cases with data; only MTS categories 3 and 4 were considered, as only one case was triaged as category 5), nor with the NACA-S score I/II vs. III/IV (*p* = 0.490, Chi^2^ test, *n* = 339 cases with data). However, we found post hoc classification as manageable in PC significantly more likely if EMS personnel considered PC treatment as appropriate (*p* = 0.002, *n* = 357 cases with data). Misclassification as eligible for redirection by EMS judgment was noted in *n* = 20 cases (5.6%), for whom hospital documentation suggested a need for the resources of an ED. Regarding first-generation migrants, misclassification did not occur more frequently (3.4%).

## Discussion

### Summary of findings

This observational study focused on low-acuity EMS patients treated in the ED and their potential for preclinical redirection to PC. Compared to previous studies by our working group that surveyed low-acuity walk-in ED patients, the cohort of low-acuity EMS patients was characterized by a higher average age, more chronic morbidity, and higher distress and subjective urgency [22]. These results are consistent with previous findings on low-acuity EMS patients [23]. ED consultations in our cohort were frequently triggered by the subjective intensity of the complaints and associated anxiety. Third parties (e.g., family members) were frequently involved in the decision to alert EMS. Only a lesser share of patients and EMS personnel classified individual medical situations as manageable in PC, respectively. Assessment of patients and EMS personnel differed significantly concerning this, judgments being based on symptoms characteristics and the resources needed. In only a quarter of the cases, EMS personnel considered diversion feasible – an assessment that included both medical and nonmedical aspects – while the respective patients concurrently expressed their theoretical openness to be diverted. EMS personnel’s assessments about redirecting were estimated as potentially incorrect in about 5% of cases. MTS triage and the NACA-S score were not significantly associated with potential eligibility for management in PC.

### Results in context

#### Determinants of ED utilization, considerations of morbidity and demographics

Almost three quarters of patients reported that their symptoms began on the day of their visit. However, 10.3% of patients alerted EMS with symptoms persisting for more than a week, which suggests a subset of patients who may have supposedly delayed seeking care – speculatively, as e.g., to access problems in ambulatory care or uncertainty about their symptoms’ severity [24, 25] – or experienced symptom exacerbation in the course of their illness. The average symptom-associated distress in our cohort was relatively high. Concurrently, half of the patients felt they needed immediate or very timely attention. In line with this, subjective distress and urgency have been reported as a major motive for low-acuity ED utilization [8, 25]. Motives for alerting EMS reported in our survey underscore the importance of subjective severity and associated anxiety as a pivotal trigger, which is in line with data from other ED settings [26–28].

This likewise applies to our data showing that third parties are quite frequently involved in a decision to alert EMS, e.g., family members or colleagues [26, 29, 30]. As our investigation of motives only captures the patients’ perspective, it does not fully represent all decision-making processes involved. Consultation decisions are multifaceted and often exceed reasoning by a single person – the patient may not even be happy about the EMS deployment but accept the realities once the ambulance arrives. However, this complexity could only be fully understood by surveying all parties involved (including relatives, etc.) or even using qualitative methods. There is little research on third-party involvement in EMS calls, but findings from nursing home settings suggest that patients’ wishes are not always considered, and some patients may potentially resign to a fate of others’ choosing [31].

Regarding health conditions, 61.9% of patients reported having at least one chronic illness, mirroring findings of chronic conditions as a potential driver of ED visits [32, 33]. Most of the study participants had regular contact with their GPs in the time before the index visit, and specialist utilization was also quite frequent, which may reflect chronic disease prevalence in this population.

Apart from chronic conditions, the high frequency of musculoskeletal complaints – including minor trauma – in our cohort might in part explain both the perceived need for ED care from the perspective of patients and others involved in alerting EMS, as a supposed need for surgical management (e.g., sutures) and radiological examinations could have been considered as beyond the capacities of a GP practice. This is reflected in the significantly lower likelihood of EMS personnel to judge such cases as ‘GP-manageable’.

A previous study investigating EMS personnel’s capacity to judge eligibility for diversion to non-hospital urgent care reported an inappropriate diversion rate of 11.6%, however this was based on agreement between EMS personnel and ED physicians [34]. In our study, misclassification that would have resulted in a diversion of patients actually in need of hospital resources was estimated as lower, but still represents a cause for concern when considering streaming patients to lower-level care. However, the occurrence of undertriage is an unresolved issue in the context of ED care, with greatly varying proportions of affected patients reported in the literature [35]. Concerning the patients classified post hoc as not eligible for PC in our study, we must stress that this was based on diagnosis and procedure data, especially radiographic imaging. It is possible that some of these cases could have been treated appropriately in a GP practice, as we did not scrutinize indications for, e.g., computed tomography in our analysis. In a hospital setting, there may be a lower threshold for ordering such measures (depending on, e.g., the experience of the treating physician), as has been described by others [36, 37].

Concerning demographic markers, our data showed that first-generation migrants were classified by EMS as redirectable with higher likelihood, which is in line with studies suggesting higher emergency care utilization for low-acuity complaints in this group [38, 39]. However, current literature rather suggests that migrants generally tend to use ambulance services less frequently [40]. Regarding this, our data does not provide further insights, as we did not study non-EMS patients comparatively. Concerning the EMS case assessments of our included patients, however, our findings do not indicate undertriage resulting from, e.g., communication barriers or ethnic prejudice, as we did not find a higher misclassification rate for this group.

#### Potential of patient redirection

As shown by our findings, the assessment of case constellations in acute care is very complex, and the opinions of patients and experts often differ. Discrepancies in opinions regarding the urgency of acute care cases – and thus the intensity of care required – are in line with previous research. In this context, several studies have compared the assessments made by EMS personnel and emergency physicians, reporting Kappa values for agreement of ~ 0.4–0.6 [34, 41, 42]. Another study highlighted marked discrepancies between triage nurses’ assessments and post hoc case reviewers, with only slight agreement (Kappa ~ 0.2) [43]. Other authors have investigated paramedics’ ability to determine the appropriate level of care or transport needed, with results suggesting caution regarding the certainty of their preclinical judgment [44]. Other results indicate that low-acuity patients may subjectively judge urgency much greater compared to professionals’ views [45].

Concerning the usefulness of severity indicators for deciding about the adequate care setting, both preclinical and clinical markers have been shown as inadequate. Especially, triage systems are not designed to decide about the adequate care setting and have proven ill-suited for identifying patients for redirection to GP care [46]. The same applies to the preclinical NACA-S score: while its categories give some indication of EMS view on potential ambulatory vs. inpatient management, this does not directly translate into feasibility of PC management, as ambulatory ED treatment can e.g., draw on the diagnostic resources of the hospital setting, while such are much more limited in a GP practice. While being widely used in EMS routine, NACA has been criticized for its subjectivity and inadequacy for scientific purposes [47]. Our results essentially underscore the inadequacy of instruments like MTS or NACA for determining eligibility for redirection to PC, as scale scores were not associated with post hoc case classification.

Scientific data on PC diversion potential of EMS cases is quite scarce, with the systematic review by Kirkland [6] identifying only four pertinent studies. In one investigation, EMS crews in a UK setting diverted low-acuity patients to a minor injury unit (MIU) based on specific complaints, but this could be realized in only 10% of potentially eligible cases, as EMS personnel conveyed patients to the ED regardless [48], highlighting the importance of their judgment beyond the existence of criteria checklists. However, the remaining EMS-based redirection projects included in the Kirkland review [6] studied special populations (older people with falls, intoxications) and thus are not readily generalizable to low-acuity cases.

In Australia, ambulance services have implemented a strategy to divert low-acuity patients identified by a commercial software tool to more suitable forms of health care, such as GPs. However, this was achieved through secondary telephone triage, which prevented the dispatch of an EMS vehicle in more than 70% of low-priority cases [49]. Notably, only 10% of EMS calls were classified as low priority in this study. In another study from the U.S., where telehealth was used to connect EMS and ED physicians for an on-site decision, non-transported patients still went to the ED by taxi in nearly 60% of the times (pre-paid rides being offered to this group), while a minority sought care at a local clinic or GP [50]. A further recent study on EMS patient diversion by Gingold et al. on a prehospital diversion program in Maryland also reported only modest reductions in ED transports [51]. Concerning judgment by different professional groups, Dale et al. interestingly found that nurses were more likely than paramedics to judge callers as not requiring an ambulance [52].

In a previous study from our hospital, prehospital triage categories and later ambulatory treatment were used as filter criteria to estimate the scope for diversion among ED utilizers, with only 1% of all ED cases classified as potentially eligible, data additionally showing discrepancies of preclinical and hospital triage [53]. Results of this study are not directly comparable to our data, as inclusion criteria differed and potential patient acceptance was not considered. In a further investigation, our working group had analyzed a cohort of EMS patients with respiratory complaints, estimating a redirection potential of 6% [23]. In only a third of these cases, patients considered PC management as a viable alternative, which is in line with our current results.

Patients, however, might underestimate the capabilities and resources of GPs, particularly in situations they perceive as urgent or anxiety-inducing. Educational efforts aimed at improving the understanding of the capacities of alternatives to emergency care – including PC – could be important to counteract this [54], especially in urban settings where the diversity of healthcare options might potentially lead to confusion about the most appropriate point of care.

This study and the available literature underline the difficulty of finding criteria for safely and effectively identifying EMS patients suitable for redirection to PC, concurrently indicating the limited quantitative potential of such schemes. Qualitative studies identified further barriers to real-world feasibility. In a recent interview study conducted by our study group, for example, EMS personnel considered PC diversion unrealistic in the context of their already high workload and limited resources [11], while Swedish researchers have stressed the importance of patient involvement in decision-making to preserve autonomy and ensure patients feeling respected [55]. The patient perspective on PC redirection by EMS, however, has been very scarcely investigated. In a previous project in our research network, we had piloted a PC linkage scheme for low-acuity ED patients without an established GP relationship, showing promising results for future GP care utilization [22]. However, this did not concern EMS callers, and no PC redirection projects are being implemented in Berlin currently.

Finally, it must be stressed that while redirection to PC may be feasible for a limited subset of low-acuity patients, the EMS system is primarily designed for high-acuity emergencies. This raises important questions about conceivable alternative pathways beyond redirection for patients who do not require EMS and ED services but still need medical care. Telehealth has shown promise in reducing unnecessary EMS transport in some settings [50, 56]. Additionally, alternative transportation services, such as medical taxis or community paramedic programs [57, 58], could provide a middle ground between full EMS deployment and patient self-transport. However, implementing such systems requires careful consideration of acceptability [59], patient safety, resource allocation, and integration with existing healthcare infrastructure. Increasing integration of EMS dispatch with the Statutory Health Insurance Physicians’ ambulatory emergency service constitutes a further important approach to better align patients’ needs and level of care in the future.

### Limitations

First and foremost, the observational study design is not suitable for investigation of causal relationships. While results provide insights into the potential scope of redirection given certain criteria (e.g., lower clinical acuity, outpatient treatment, patients’ openness to alternative care and professionals’ assessment as feasible), redirection feasibility in the real world would depend on many factors not represented in our data, like capacity and willingness of GP practices to accept EMS patients. As discussed, qualitative findings from EMAPREPARE have shed doubt on EMS acceptance in this context [11].

We must also stress that our data does not represent shares of patients potentially eligible for redirection among unselected ED patients – or all EMS patients – as implicit to having investigated outpatient cases only. This limits comparability to other studies using different inclusion criteria.

The survey also did not assess patients’ awareness of alternative emergency care options, such as the Statutory Health Insurance Physicians’ ambulatory emergency service. Such could have provided additional insights into their decision-making.

Furthermore, it is possible that our findings are influenced by characteristics of the German healthcare system, as e.g., PC capabilities (e.g., imaging) may vary considerably across different healthcare systems [60, 61]. Additionally, the demographics, social structure and health care environment of the highly urbanized setting of central Berlin might have shaped participants’ expectations and perceptions of healthcare services, so results could not be fully applicable to other geographical or socio-economic environments. Studies have demonstrated markedly higher rates of EMS utilization in urban areas [62], with Berlin exhibiting particularly elevated numbers [63]. Besides socio-demographic factors, potentially stronger PC relationships in rural settings leading patients to more frequently consider alternative care options may play a role here.

Another key limitation of this pilot study is its relatively small sample size, and the potential influence of the COVID-19 pandemic on emergency care utilization and delivery during the study period (2021–2024) must be noted. Especially in its first phases, our study experienced considerably delayed recruitment due to pandemic-related workflow restrictions in EDs, temporary institutional suspensions of non-vital studies, and ensuing study staffing limitations. The considerable length of the survey additionally posed challenges, as the inclusion process and face-to-face data collection required substantial time and personnel resources. Moreover, the pandemic situation may have altered healthcare seeking behaviors.

## Conclusions

By triangulation of a patient survey, EMS personnel case evaluations, and treatment documentation, this study provides multifaceted insights into the potential for and barriers to redirecting eligible low-acuity patient populations from EMS to GP practices.

Realistically, PC diversion might be achieved in no more than a quarter of cases finally being treated as ED outpatients. However, this must be regarded with due caution, as treatment data in a share of these cases suggest potential misclassification, and fundamental acceptance of redirection schemes by involved stakeholders is doubtful (EMS personnel) or has not been studied (GPs).

Our analysis of various potential markers for identifying low-acuity EMS patients suitable for PC diversion has yielded inconclusive results. Marked discrepancies between patient self-assessment, paramedic judgment, and clinical severity were observed. Indices like MTS triage and NACA scores highlight the complexity of accurately determining patient acuity and appropriate care pathways. No single marker emerged as suitable for determining EMS patient disposition, underscoring the multifaceted nature of medical decision-making in prehospital settings. The lack of concordance between assessments emphasizes the need for a more comprehensive and nuanced approach. Before implementing diversion schemes, it is imperative to develop and rigorously validate new composite measures that can more accurately identify eligible patients. These should ideally incorporate multiple factors and be capable of navigating the inherent variability in patient presentation and healthcare needs.

Considering the limited scope for PC redirection, future research should also explore the potential of telemedicine care options and alternative transportation methods to address the gap in service provision for patients who do not require EMS and ED care but still need medical attention.

## Data Availability

The dataset used and analyzed during this study is available from the corresponding author on reasonable request.

## Abbreviations

ED: Emergency Department
PC: Primary Care
GP: General Practitioner
EMAPREPARE: Emergency and Acute Medicine – Primary Care Demands in Patients resorting to Emergency Departments
MTS: Manchester Triage System
EMANet: Emergency and Acute Medicine Network for Health Care Research Berlin
CASMIN: Comparative Analysis of Social Mobility in Industrial Nations
PHQ: Patient Health Questionnaire
SD: Standard Deviation
